# FNeXter: A Multi-Scale Feature Fusion Network Based on ConvNeXt and Transformer for Retinal OCT Fluid Segmentation

**DOI:** 10.3390/s24082425

**Published:** 2024-04-10

**Authors:** Zhiyuan Niu, Zhuo Deng, Weihao Gao, Shurui Bai, Zheng Gong, Chucheng Chen, Fuju Rong, Fang Li, Lan Ma

**Affiliations:** Tsinghua Shenzhen International Graduate School, Tsinghua University, Shenzhen 518055, China; niuzy21@mails.tsinghua.edu.cn (Z.N.); dz20@mails.tsinghua.edu.cn (Z.D.); gwh20@mails.tsinghua.edu.cn (W.G.); bsr22@mails.tsinghua.edu.cn (S.B.); gz20@mails.tsinghua.edu.cn (Z.G.); chenchch94@sz.tsinghua.edu.cn (C.C.); rongfuju@sz.tsinghua.edu.cn (F.R.); li.fang@sz.tsinghua.edu.cn (F.L.)

**Keywords:** retinal fluid segmentation, Transformer, optical coherence tomography, attention

## Abstract

The accurate segmentation and quantification of retinal fluid in Optical Coherence Tomography (OCT) images are crucial for the diagnosis and treatment of ophthalmic diseases such as age-related macular degeneration. However, the accurate segmentation of retinal fluid is challenging due to significant variations in the size, position, and shape of fluid, as well as their complex, curved boundaries. To address these challenges, we propose a novel multi-scale feature fusion attention network (FNeXter), based on ConvNeXt and Transformer, for OCT fluid segmentation. In FNeXter, we introduce a novel global multi-scale hybrid encoder module that integrates ConvNeXt, Transformer, and region-aware spatial attention. This module can capture long-range dependencies and non-local similarities while also focusing on local features. Moreover, this module possesses the spatial region-aware capabilities, enabling it to adaptively focus on the lesions regions. Additionally, we propose a novel self-adaptive multi-scale feature fusion attention module to enhance the skip connections between the encoder and the decoder. The inclusion of this module elevates the model’s capacity to learn global features and multi-scale contextual information effectively. Finally, we conduct comprehensive experiments to evaluate the performance of the proposed FNeXter. Experimental results demonstrate that our proposed approach outperforms other state-of-the-art methods in the task of fluid segmentation.

## 1. Introduction

The macula is located at the center of the retina, responsible for human vision and color perception. Macular edema is a swelling in a portion of the retina, caused by the accumulation of fluid that has leaked from damaged retinal vessels. This condition is usually a result of retinal diseases such as age-related macular degeneration (AMD), retinal vein occlusion (RVO), or diabetic macular edema (DME). The primary types of retinal fluid causing macular edema include intraretinal fluid (IRF), subretinal fluid (SRF), and pigment epithelial detachment (PED). Macular edema can disrupt the normal structure of the retina, leading to vision impairment or even blindness, making it one of the most common causes of vision loss worldwide [1].

Optical coherence tomography (OCT) is a non-contact, high-resolution imaging technique with micron-level accuracy [2]. OCT has been widely used in the diagnosis of retinal diseases and is the standard clinical method for observing and evaluating retinal fluid in the macular region. For the precise diagnosis of retinal diseases, the development of personalized treatment strategies, and the evaluation of therapeutic effectiveness, it is essential to conduct an accurate segmentation and quantitative analysis of the retinal fluid in the macular region. The process of manual segmentation of retinal fluid is labor-intensive, time-consuming, and prone to individual biases and potential errors. Given these challenges, there is a compelling necessity for the exploration of computer-aided automatic segmentation methodologies.

There has been extensive research on the computer-aided automatic segmentation of OCT fluid. Traditional automated segmentation methods have predominantly relied on image processing algorithms, such as directional graph search [3] and level set [4] methods, or machine learning methods using manually extracted features [5]. However, these techniques often exhibit limited performance and poor generalization, failing to meet clinical requirements. With the advancement of machine learning, deep-learning-based approaches have been increasingly applied to the task of fluid segmentation and have achieved promising results. However, several challenges remain unresolved.

Medical image segmentation tasks are often designed for certain types of images, and leveraging the inherent prior knowledge can be significantly advantageous for developing high-performance segmentation models. Retinal fluid lesions predominantly occur in the central region of OCT images. However, most existing research does not capitalize on this prior knowledge, leading to insufficient focus on the lesion areas. There is a lack of cost-effective, end-to-end automated methods to guide the model’s attention towards the location of the lesions. Therefore, we propose the region-aware spatial attention (RASA) module, introducing prior knowledge of lesion locations, thereby enhancing the model’s capability to extract lesion features. Furthermore, due to the uncertainty of fluid leakage and accumulation, there is significant variability in the shape, location, and size of fluid regions, often with complex and curved boundaries. Additionally, the low contrast and presence of noise in OCT images may result in blurred or ambiguous boundaries. Hence, the model requires robust multi-scale feature extraction capabilities to identify complex lesions. Confronted with the task of segmenting lesions with various scales, existing models exhibit limited capability in aggregating multi-scale features. Consequently, we propose the self-adaptive multi-scale feature fusion attention module, which fuses and extracts multi-scale features from adjacent encoder stages, enhancing the model’s ability to acquire global multi-scale contextual information. Furthermore, current methods for fluid segmentation predominantly rely on CNN-based U-shaped architectures. However, CNN-based approaches are limited in their capacity to capture long-range dependencies. On the contrary, the Multi-Head Self-Attention (MSA) in Transformer has shown excellent performance in modeling non-local similarities and long-range dependencies. As a result, we propose a multi-scale hybrid encoder module that integrates both the Convolutional Neural Network ConvNeXt and Transformer, leveraging the strengths of both to comprehensively extract local detail information and global features.

Our main contributions can be summarized as follows:

- We design a novel global multi-scale hybrid encoder module, integrating ConvNeXt, Transformer, and region-aware spatial attention(RASA). This module can simultaneously capture long-range and short-range dependencies while possessing adaptive spatial region-aware capabilities.
- We introduce a new self-adaptive multi-scale feature fusion attention (SMFFA) module to extract fusion features adaptively at the skip connections.
- We conduct extensive experiments on public datasets to validate the performance of our model. The results demonstrate that our model outperforms other methods, achieving state-of-the-art performance.

## 2. Related Work

### 2.1. Fluid Segmentation

In recent years, researchers have developed a series of image segmentation models based on deep learning technology, such as U-Net [6], FCN [7], Seg-Net [8], and Deeplabv3+ [9]. Adapting to the unique requirements of medical imaging, various adaptations and enhancements of these models have been proposed, with a focus on segmenting specific organs, structures, and lesions. Given that U-Net has demonstrated exceptional performance in medical image segmentation tasks, most contemporary medical image segmentation models are refined versions based on U-Net, such as U-Net++ [10], Attention U-Net [11], ResUnet [12], and nnU-Net [13]. Alongside these developments, there have been diverse methods proposed specifically for the segmentation of fluid in OCT images.

Lu et al. [14] incorporated fluid spatial information from retinal layer segmentation and employed random forest classification as a post-processing method to address false-positive issues, achieving first place in the RETOUCH challenge. This methodology employed both pre-processing and post-processing techniques, enhancing the accuracy of lesion segmentation. Beyond this, researchers have proposed a variety of methods incorporating pre-processing and post-processing techniques to refine the segmentation process [15,16,17]. Pre-processing methods [16], such as denoising and layer segmentation, serve to augment the input data for the segmentation models. Post-processing strategies using machine learning techniques [15] further reduce the occurrence of false positives. While these strategies collectively enhance the efficacy of segmentation algorithms, it is noteworthy that pre-processing and post-processing can introduce potential information loss, augment computational demands, and add to the overall complexity of the process. With the advancements in model architectures and attention mechanisms, the capability of models to extract lesion features has been significantly enhanced. Contemporary research predominantly harnesses attention mechanisms to bolster information extraction, thereby reducing the need for additional processing steps. Consequently, most current methodologies employ end-to-end pipelines for retinal fluid segmentation, simplifying the process.

Hu et al. [18] proposed a segmentation model leveraging stochastic atrous spatial pyramid pooling (sASPP). This model employed dilated convolutions to efficiently extract multi-scale pathological features, aiming to enhance segmentation accuracy while reducing the risk of overfitting. Feng et al. [19] proposed the CPFNet, a model that incorporated two multi-scale pyramid modules. This design facilitated the fusion of global contextual information and demonstrated superior performance in specific tasks, such as retinal macular segmentation. Liu et al. [20] utilized attention gates to process features from dense skip connections and incorporated regression loss to address the issue of erroneous merging of retinal fluid regions. Xing et al. [21] proposed a curvature loss function, specifically designed by incorporating shape prior knowledge of the fluid, which consequently elevated the precision of shape and boundary delineation.

### 2.2. Vision Transformer

In recent times, the introduction and adaptation of the Transformer architecture in computer vision have led to notable breakthroughs. Transformer-based approaches have achieved state-of-the-art (SOTA) performance across a wide array of visual tasks [22,23], etc. The Vision Transformer (ViT) [24] represented the pioneering effort of integrating the Transformer framework into image classification. It converted the input image into a series of discrete patches, subsequently deploying multi-head self-attention mechanisms for processing. The Swin Transformer [25] divided the input image into multiple non-overlapping windows and employed a shifted window-based self-attention mechanism, reducing computational complexity and achieving superior results.

Given the outstanding performance of Transformer in natural image tasks, numerous studies have explored the use of Transformer in the construction of medical image segmentation models. In previous studies, Transformers have been employed both as components [26] within segmentation models and as independent architectures [27] for segmentation. TransUNet [26] was a hybrid framework that melded Convolutional Neural Network (CNN) and Transformer, capitalizing on the strengths of both to achieve comprehensive feature extraction. SwinUNet [27] represented the first model to construct a U-Net architecture entirely based on Transformer, offering advantages in capturing long-range dependency information. Huang et al. [28] proposed the MISSFormer model, which innovatively refined the feed-forward network within the Transformer and incorporated a remixed Transformer context bridge in the skip connection. This design sought to explore both global dependencies and local contexts, ensuring a more holistic feature extraction. Wang et al. [29] introduced UCTransNet, a model that replaced traditional skip connection with a Transformer-based multi-scale channel-wise cross attention. This design facilitated the amalgamation of multi-scale channel information, ensuring that the model captured more sophisticated channel dependencies. However, Transformer architectures encounter the challenge of requiring vast amounts of annotated data. Moreover, Transformer architectures primarily focus on extracting global features, which is often insufficient for medical image segmentation tasks. There are many minor lesions in OCT images, necessitating the extraction of detailed local features. Consequently, the crux of research lies in integrating both CNN and Transformer models, with explorations into how to effectively fuse the multi-scale features obtained.

## 3. Methodology

In this section, we first provide an overview of the proposed method. Subsequently, we present the hybrid encoder incorporating ConvNeXt Transformer and region-aware spatial attention module. Further, we introduce the self-adaptive multi-scale feature fusion attention module. Lastly, we delineate the components of the loss function.

### 3.1. Overview

**Figure 1:**
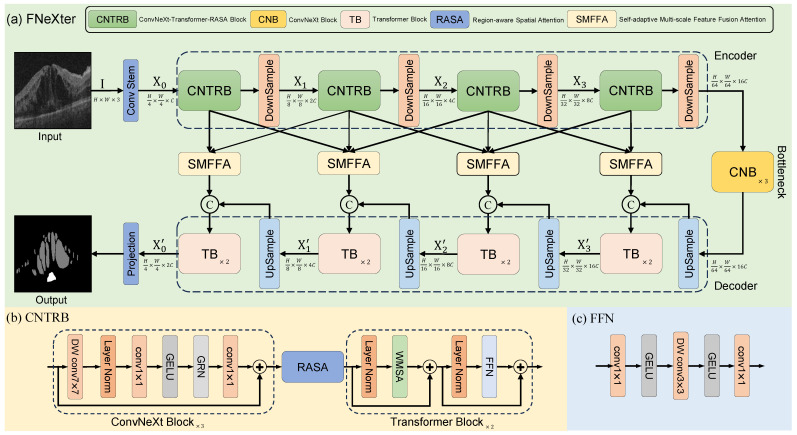
Overall architecture of our proposed FNeXter for retinal fluid segmentation. (**a**) FNeXter adopts a U-shaped structure, composed of an encoder, a bottleneck, and a decoder. Both the encoder and decoder consist of four stages. In the encoder, each stage contains a CNTRB (ConvNeXt-Transformer-RASA Block), while in the decoder, each stage is equipped with two Transformer blocks. The bottleneck layer includes three ConvNeXt blocks. The SMFFA module is utilized to fuse multi-scale features, thereby enhancing the skip connections between the corresponding stages of the encoder and decoder. (**b**) Within the encoder, the structure of CNTRB is formed by ConvNeXt blocks, Transformer blocks, and region-aware spatial attention (RASA). (**c**) The feed-forward network of the Transformer block consists of two 1×1 convolutional layers, two GELU activation layers, and a depth-wise 3×3 convolutional layer.

The architecture of the model is depicted in Figure 1, consisting of an encoder, a bottleneck, and a decoder. Specifically, given an input image $I\in R^{H\times W\times 3}$, where *H*, *W*, and 3 denote the height, width, and channel count, respectively, the image is first processed through a Convolutional Neural Network module termed stem. This includes a 4×4 convolutional layer with a stride of 2, serving to extract initial features and implement downsampling. Consequently, this produces a feature map of a resolution quartered from the original image, having a channel dimension, C, of 96. It can also be denoted as $I\in R^{\frac{H}{4}\times \frac{W}{4}\times C}$.

Subsequently, the feature map undergoes progressive deep feature extraction via four encoder stages. Each stage consists of a ConvNeXt module, a Transformer module, and a region-aware spatial attention module. After each encoder stage, a downsampling layer composed of LayerNorm and a 2×2 convolutional layer with a stride of 2 is utilized. This serves to halve the spatial dimensions of the feature map and double the channel count. Consequently, the feature representation from the ith stage in the encoder is given as $X_{i}\in R^{\frac{H}{2^{i+2}}\times \frac{W}{2^{i+2}}\times 2^{i}C}$ where i∈{0,1,2,3} indexes the four stages. Thirdly, the feature maps pass through a bottleneck layer composed of three ConvNeXt blocks, where further feature extraction and combination take place. Subsequently, the feature maps from the bottleneck layer are fed into the decoder section for continued feature extraction and upsampling operations. Each stage of the decoder is made up of two Transformer blocks. After each decoder stage, the feature maps are processed through an upsampling layer, which employs bilinear interpolation followed by a 3×3 convolutional operation, doubling the spatial dimensions while halving the channel count. We also employ a self-adaptive multi-scale feature fusion attention module to enhance the skip connections, allowing for a better fusion of multi-scale features from adjacent encoder stages while preserving both global and local information. Finally, a 1×1 convolutional layer is used to generate the segmentation results.

### 3.2. ConvNeXt-Transformer-RASA Block

Convolutional Neural Networks (CNNs) possess local perceptual properties, enabling them to extract features from local regions of input data and thereby capturing an image’s local structure and information. Moreover, they benefit from an inductive bias inherent in their architecture. This bias leans the network towards learning specific functions, such as translational invariance, crucial for image processing tasks. However, CNNs have certain limitations in modeling long-range dependencies. In contrast, the Transformer addresses this shortcoming by employing window shift operations and multi-head self-attention mechanisms, facilitating the capture of interdependent relationships across different regions of an image. Additionally, we introduce a region-aware spatial attention (RASA) module that offers an added layer of spatial understanding by focusing on critical areas within the image. The RASA module provides the model with prior knowledge of lesion locations, emphasizing regions with lesions and de-emphasizing background areas according to their contextual significance. By integrating these three distinct yet complementary modules into a single encoder stage, we achieve a richer and more robust feature representation. This hybrid architecture capitalizes on the local feature extraction strengths of ConvNeXt, the long-range dependency handling of the Transformer, and the context-sensitive region-awareness introduced by the RASA module. Collaboratively, they contribute to a more comprehensive understanding of both local and global characteristics of the image. The specific structure of the encoder is illustrated in Figure 1b.

#### 3.2.1. ConvNeXt

Convolutional Neural Networks (CNNs) have been demonstrated to efficiently encode local spatial details. They are also more conducive to training. Consequently, CNNs are employed in our encoder. To further harness the strengths of CNNs and integrate the benefits of the latest advancements in Transformer architecture, researchers have proposed the ConvNeXt architecture [30], which includes Inverted Bottleneck and large kernels. Woo et al. [31] extend the self-supervised pre-training methodology to the ConvNeXt architecture and introduce a novel global response normalization (GRN) layer, leading to the development of the ConvNeXt-V2 model. This enhanced model demonstrates superior performance in both image classification and semantic segmentation tasks. In our encoder, we employ basic ConvNeXt-V2 blocks for feature extraction. As depicted in Figure 1b, the ConvNeXt-V2 block consists of a depth-wise convolution with kernel size of 7×7, a layer normalization, a dimension-expansion pointwise convolution (1×1 convolution layer) with a GELU activation, a novel global response normalization and a dimension-reduction pointwise convolution. For the first pointwise convolution layer, we set the expansion ratio to 4. Similarly, the dimension reduction ratio of the subsequent pointwise convolution is set to 4 for feature recovery. The specific implementation formula is as follows:

(1) $$\begin{array}{c} F^{\prime }=\mathrm{LN}\left({\mathrm{DWConv}}_{7\times 7}\left(F_{in}\right)\right), \end{array}$$

(2) $$\begin{array}{c} F_{out}={\mathrm{Conv}}_{1\times 1}\left(\mathrm{GRN}\left(\mathrm{GELU}\left({\mathrm{Conv}}_{1\times 1}\left(F^{\prime }\right)\right)\right)\right)+F_{in}, \end{array}$$

where $F_{in}$ represents the input feature of ConvNeXt block. $F^{\prime }$ denotes the output feature from depth-wise convolution layer. $F_{out}$ denotes the final output. LN(·) represents the layer normalization, while GELU and GRN refer to the non-linear activation function and global response normalization, respectively. In our ConvNeXt block, the inductive bias inherent in the convolutional operations complements the subsequent Transformer block, facilitating easier training. By employing large-kernel convolutional layers, the model significantly expands its receptive field, which is crucial for capturing more extensive contextual information. This expansion plays a pivotal role in augmenting the model’s capabilities for learning global long-range representations, enabling it to better understand and process data in tasks that require a broader view of context. Furthermore, these convolutional operations are adept at capturing local fine-grained details, enabling our ConvNeXt to achieve a comprehensive understanding of both global and local feature representations.

#### 3.2.2. Transformer Block

As illustrated in Figure 1b,c, the architecture of the Transformer block consists of a window-based multi-head self-attention(WMSA), two layer normalization operations, and a feed-forward neural network (FFN). Emulating the approach of the Swin Transformer [25], we incorporate Window Shift Operations (WSO) into the Window-based Multi-head Self-Attention Block (WMSA) to introduce cross-window connections. The Transformer block is capable of further modeling global long-range dependencies and non-local similarities on top of the ConvNeXt foundation. The Transformer block can be expressed as follows:

(3) $$\begin{array}{c} F^{\prime }=\mathrm{WMSA}\left(\left(\mathrm{LN}\left(F_{in}\right)\right)\right)+F_{in}, \end{array}$$

(4) $$\begin{array}{c} F_{out}=\mathrm{FFN}\left(\mathrm{LN}\left(F^{\prime }\right)\right)+F^{\prime }, \end{array}$$

where $F_{in}$ and $F_{out}$ represent the input and output feature maps of the Transformer block, respectively. LN(·) represents the layer normalization. WMSA refers to the Window-based Multi-head Self-Attention, which computes the interactions among tokens within each window. The input feature map is first partitioned into non-overlapping windows, each of size L×L. Subsequently, the features $X\in R^{L\times L\times C}$ of each window are flattened and transposed, and then linearly projected into query Q, key K, and value $V\in R^{L^{2}\times C}$.

(5) $$\begin{array}{c} Q=X_{in}W_{Q},K=X_{in}W_{K},V=X_{in}W_{V}, \end{array}$$

where $W_{Q},W_{K},W_{V}\in R^{C\times C}$ are learnable parameters, representing the projection matrices for query, key, and value, respectively. We then split Q,K, and V into *k* heads along the channel dimension as $Q=[Q_{1},\ldots ,Q_{k}]$, $K=[K_{1},\ldots ,K_{k}]$, and $V=[V_{1},\ldots ,V_{k}]$. The dimension for each head is $d_{k}=\frac{C}{k}$. The Self-Attention (SA) mechanism for the *j* head is formulated as follows:

(6) $$\mathrm{SA}\left(Q_{j},K_{j},V_{j}\right)=\mathrm{softmax}\left(\frac{Q_{j}K_{j}^{T}}{\sqrt{d_{k}}}\right)V_{j}$$

where $Q_{j},K_{j},$ and $V_{j}$ denote the query, key, and value for the *j* head, respectively. The output tokens $X_{o}\in R^{L^{2}\times C}$ for each window can be obtained by the equation

(7) $$X_{o}=\overset{k}{\underset{j=1}{\mathrm{Concat}}}\left(\mathrm{SA}\left(Q_{j},K_{j},V_{j}\right)\right)W_{O}+B\;$$

where Concat(·) indicates the concatenation operation, $B\in R^{L^{2}\times C}$ represents the positional embedding, and $W_{O}\in R^{C\times C}$ are learnable parameters. The output tokens $X_{o}$ are then reshaped to produce the output feature map $X_{\mathrm{out}}\in R^{L\times L\times C}$. Finally, the output features from all the windows are aggregated to form the final output feature map.

#### 3.2.3. Region-Aware Spatial Attention

**Figure 2:**
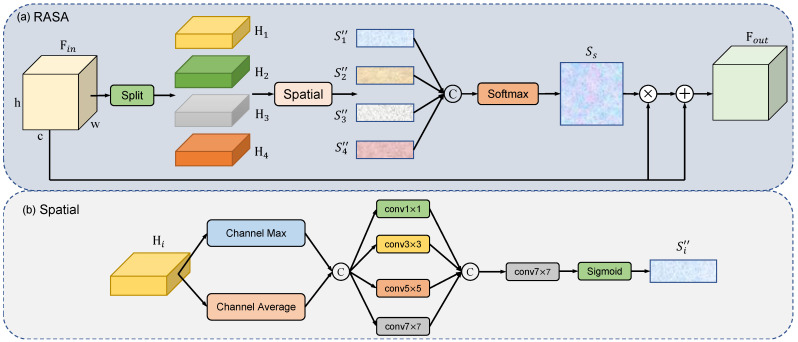
(**a**) Overall architecture of the region-aware spatial attention (RASA) module. (**b**) The detailed structure of the spatial attention in the region-aware spatial attention (RASA) module.

To selectively emphasize the lesion area based on its contextual importance, we introduce the region-aware spatial attention (RASA) module for the incorporation of lesion location prior knowledge. The steps of our spatial attention are delineated as follows: As illustrated in Figure 2a, for the input feature map $F_{in}\in R^{H\times W\times C}$, we divide it into four equal parts $H_{i}\in R^{\frac{H}{4}\times W\times C}$ in a top-down sequence, where i∈{1,2,3,4} represents four segments.

(8) $$H_{i}=\mathrm{Split}\left[F_{in}\right],\;i\in \left\{1,2,3,4\right\},$$

where Split denotes the division of the feature map into four equal parts along the height dimension, in a top-down sequence. Subsequently, as illustrated in Figure 2b, we apply spatial attention to each of the four segments individually. For each feature map of segment $H_{i}$, we compute both the average and maximum values in the channel dimension, resulting in two tensors. These two tensors are then concatenated along the channel dimension to obtain $S_{i}^{\prime }\in R^{\frac{H}{4}\times W\times 2}$.

(9) $$S_{i}^{\prime }=\mathrm{Concat}\left[{\mathrm{GAP}}^{c}\left(H_{i}\right),{\mathrm{GMP}}^{c}\left(H_{i}\right)\right)],\;i\in \left\{1,2,3,4\right\},$$

where Global Average Pooling (${\mathrm{GAP}}^{c}$) and Global Maximum Pooling (${\mathrm{GMP}}^{c}$) represent the computed average and maximum values along the channel dimension, respectively. Following this, we apply four convolutional layers with kernel sizes of 1×1, 3×3, 5×5, and 7×7, respectively, to the concatenated tensor $S_{i}^{\prime }$, aiming to capture multi-scale information. As a result, we obtain four tensors containing information at different scales. To further facilitate fusion and attention computation, we concatenate these tensors along the channel dimension. Following the concatenation, a 7×7 convolutional layer is employed to reduce the channel dimension of the concatenated output from 4 to 1. Finally, the attention weights for each segment are generated using a sigmoid function.

(10) $$S_{i}^{\prime \prime }=\sigma \left({\mathrm{Conv}}_{7\times 7}\left(\overset{1,3,5,7}{\underset{j}{\mathrm{Concat}}}\left[{\mathrm{Conv}}_{j\times j}S_{i}^{\prime }\right]\right)\right),\;i\in \left\{1,2,3,4\right\},$$

where Concat denotes the concatenation of tensors processed by the four convolutional layers along the channel dimension. σ represents sigmoid activation function. Lastly, the attention weights obtained for the four segments are concatenated along the height dimension and normalized using a softmax function, yielding a final 2D spatial attention map $S_{s}$. This map is then element-wise multiplied with the given input feature map $F_{in}$ to produce the weighted features $F_{out}$.

(11) $$\begin{array}{c} S_{s}=\mathrm{softmax}\left(\mathrm{Concat}\left[S_{1}^{\prime \prime },S_{2}^{\prime \prime },S_{3}^{\prime \prime },S_{4}^{\prime \prime }\right]\right), \end{array}$$

(12) $$\begin{array}{c} F_{out}=S_{s}⊗F_{in}, \end{array}$$

The softmax normalization ensures that the model gives weight to each area based on its relative importance when fusing information from the four distinct regions. This adaptive weighting allows the model to recognize the significance of different spatial positions. Fluid lesions typically appear in the central region of OCT images, which constitutes our prior knowledge of lesion locations. Through our proposed region-aware spatial attention, we can incorporate this lesion location prior into the model, adaptively guiding the model to focus on the central region where the lesions are located. This approach effectively captures lesion-related information, thereby enhancing the model’s awareness of region and lesion. Moreover, during the spatial attention computation process, we employ convolutional layers with four distinct kernel sizes. This strategy effectively expands the receptive field, enabling the model to extract multi-scale spatial information more efficiently.

### 3.3. Self-Adaptive Multi-Scale Feature Fusion Attention

**Figure 3:**
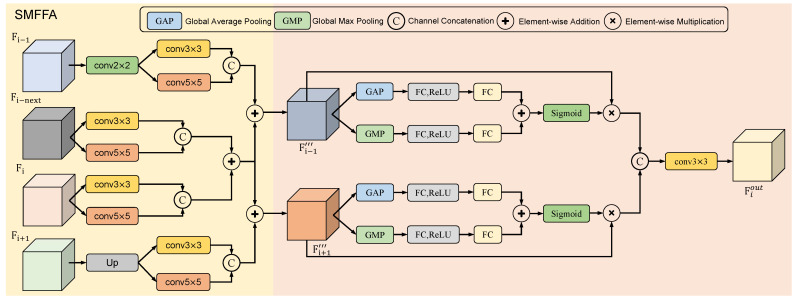
The detailed structure of the self-adaptive multi-scale feature fusion attention (SMFFA) module.

In the U-Net architecture, the output from each stage of the encoder is concatenated with the output from the corresponding stage of the decoder, an operation commonly referred to as skip connection. The skip connections allow for the integration of low-level and high-level features, mitigating information loss and enhancing the model’s performance. To further integrate multi-scale features and enhance the model’s ability to learn global contextual information, we introduce the self-adaptive multi-scale feature fusion attention (SMFFA). Many researchers have proposed methods for feature fusion, such as the hierarchical attention module (HAM) introduced by Tao et al. [32], which employs different fusion methods to integrate feature maps of varying channel-spatial ratios to learn discriminative features. Differently from the SAM, our SMFFA approach fuses a broader range of multi-scale features, enhancing multi-scale information and diverse features. Additionally, we enhance spatial features in the feature extraction encoder stages, and in SMFFA, further augment important multi-scale features through attention mechanisms. Building upon the foundation of skip connections, SMFFA is capable of adaptively fusing and extracting multi-scale features from adjacent stages. The detailed definition is as follows: For the output feature map of each encoder stage $F_{i}$ where i∈{1,2,3,4} indexes the four stages. As illustrated in Figure 3, for feature $F_{i}$, we merge it with the feature of its neighboring stage. For the SMFFA of the second stage and third stage, the feature is fused with the features from both the preceding and the succeeding stages. However, for the SMFFA of the first stage and fourth stage, the feature is only fused with the feature from its immediate neighboring stage.

For the SMFFA at encoder stage *i* where i∈{1,2,3,4}, we fuse the current stage feature $F_{i}$ and additional feature $F_{i-next}$, the preceding stage feature $F_{i-1}$, and the succeeding stage feature $F_{i+1}$. To better leverage the advantages of the encoder architecture and fuse features across multiple scales and layers, we introduce an additional feature $F_{i-next}$ extracted right after the ConvNeXt module of the current encoder stage, in addition to the output feature $F_{i}$ at each stage. This strategy aims to harness the strengths of both the ConvNext and Transformer modules in feature processing. For the preceding stage feature $F_{i-1}$, we apply a convolutional layer with a kernel size of 2×2 and a stride of 2 to perform downsampling, halving the spatial resolution of the feature map. For the succeeding stage feature $F_{i+1}$, we employ bilinear interpolation to perform upsampling, doubling the spatial size of the feature map.

(13) $$F_{i-1}^{\prime }={\mathrm{Conv}}_{2\times 2}\left(F_{i-1}\right),$$

(14) $$F_{i+1}^{\prime }=\mathrm{UP}\left(F_{i+1}\right),$$

To effectively integrate multi-scale features, we employ convolutional layers with kernel sizes of 3×3 and 5×5 to process the aforementioned four features. For feature $F_{i}\in R^{H\times W\times C}$, we apply both layers to $F_{i}$ yielding two features each with channel dimension reduced to $\frac{C}{2}$. These are then concatenated along the channel dimension to produce $F_{i}^{\prime }$. Similar operations are applied to $F_{i-next}$ to obtain $F_{i-next}^{\prime }$. Likewise, $F_{i-1}^{\prime }$ and $F_{i+1}^{\prime }$ are processed to obtain $F_{i-1}^{\prime \prime }$ and $F_{i+1}^{\prime \prime }$, respectively.

(15) $$F_{m}^{\prime }=\mathrm{Concat}\left({\mathrm{Conv}}_{3\times 3}\left(F_{m}\right),{\mathrm{Conv}}_{5\times 5}\left(F_{m}\right)\right),$$

where $F_{m}$ represents one of the several features described previously, specifically belonging to the set {$F_{i},F_{i-next},F_{i-1}^{\prime },F_{i+1}^{\prime }$}. Meanwhile, $F_{m}^{\prime }$ denotes the output feature map after processing, and belongs to the set {$F_{i}^{\prime },F_{i-next}^{\prime },F_{i-1}^{\prime \prime },F_{i+1}^{\prime \prime }$}. By employing these two convolutional layers with kernel sizes of 3×3 and 5×5, the model is further enabled to capture features across multiple scales, allowing the network to recognize both fine-grained and coarser patterns within OCT images. Additionally, this operation enhances the model’s ability to capture more contextual information, providing a more comprehensive representation of the data and enriching the feature space. Subsequently, for the current encoder stage, the concatenated features $F_{i}^{\prime }$ and $F_{i-next}^{\prime }$ undergo element-wise addition to yield the final feature $F_{i}^{\prime \prime }$ for the current stage.

(16) $$F_{i}^{\prime \prime }=F_{i}^{\prime }⊕F_{i-next}^{\prime },$$

where ⊕ denotes element-wise addition. Subsequently, the processed feature of the current stage, $F_{i}^{\prime \prime }$, is element-wise added to the features of the adjacent preceding and succeeding stages, $F_{i-1}^{\prime \prime }$ and $F_{i+1}^{\prime \prime }$, respectively. The sum of $F_{i}^{\prime \prime }$ and $F_{i-1}^{\prime \prime }$ yields a preliminarily fused multi-scale feature $F_{i-1}^{\prime \prime \prime }$. Similarly, the sum of $F_{i}^{\prime \prime }$ and $F_{i+1}^{\prime \prime }$ results in another preliminarily fused multi-scale feature $F_{i+1}^{\prime \prime \prime }$. By subjecting four features from adjacent stages to convolutional and concatenation operations, the model gains enhanced multi-scale feature representation. This not only allows for the more precise recognition of detailed information but also aids in capturing global contextual information, thereby preserving semantic richness across different scales and rendering a more comprehensive feature representation.

(17) $$F_{i-1}^{\prime \prime \prime }=F_{i-1}^{\prime \prime }⊕F_{i}^{\prime \prime },$$

(18) $$F_{i+1}^{\prime \prime \prime }=F_{i}^{\prime \prime }⊕F_{i+1}^{\prime \prime },$$

To better process the fused features, we utilize channel attention to further enhance important multi-scale features. For the fused feature $F_{i-1}^{\prime \prime \prime }$, we first apply Global Average Pooling (GAP) and Global Max Pooling (GMP) operations. The outputs from these operations are then passed to respective fully connected layers. These are subsequently processed through a ReLU activation function and another fully connected layer, enabling the model to learn a compact representation. Finally, the processed features from both paths are summed and passed through a sigmoid activation function to learn attention weights. The attention weights are element-wise multiplied with the input fused feature to obtain the attention-modulated feature $F_{i-1}^{c}$. Through this attention module, essential features within the fused representation are further emphasized. This enables the model to adaptively learn and selectively extract salient channel features, thereby enhancing the specificity of feature extraction.

(19) $$F_{i-1}^{cavg}=W_{2}\left(\mathrm{ReLU}\left(W_{1}\mathrm{GAP}\left(F_{i-1}^{\prime \prime \prime }\right)\right)\right),$$

(20) $$F_{i-1}^{cmax}=W_{2}\left(\mathrm{ReLU}\left(W_{1}\mathrm{GMP}\left(F_{i-1}^{\prime \prime \prime }\right)\right)\right),$$

(21) $$F_{i-1}^{c}=\sigma \left(\left(F_{i-1}^{cavg}⊕F_{i-1}^{cmax}\right)\right)⊗F_{i-1}^{\prime \prime \prime },$$

where $W_{1}$ and $W_{2}$ denotes fully connected layer. σ denotes sigmoid activation function. ⊗ denotes element-wise multiplication. Similarly, the feature $F_{i+1}^{\prime \prime \prime }$ undergoes attention processing as described in the above equations, resulting in an attention-modulated feature represented by $F_{i+1}^{c}$. Ultimately, we concatenate the two attention-enhanced fused features and pass them through a 3×3 convolutional layer for dimension reduction, yielding the final fused feature $F_{i}^{out}$, which is then relayed to the corresponding stage of the decoder.

(22) $$F_{i}^{out}={\mathrm{Conv}}_{3\times 3}\left(\mathrm{Concat}\left(F_{i-1}^{c},F_{i+1}^{c}\right)\right),$$

As depicted in Figure 3, the aforementioned operations collectively form our self-adaptive multi-scale feature fusion attention (SMFFA). Through our proposed SMFFA, we have enhanced the traditional skip connections by merging distinct features from adjacent encoder stages. Firstly, within the current encoder stage, we fuse the features processed by ConvNeXt with the final output features of the entire stage. This operation facilitates multi-level feature extraction, as ConvNeXt primarily focuses on local, detailed features, while the final stage output captures more global and high-level semantic information. This enhances the model’s robustness to various types of data. Secondly, all features from adjacent stages undergo processing through convolutional layers with different kernel sizes, preserving semantic richness across multiple scales. Furthermore, our SMFFA effectively fuses multi-scale and multi-level features across multiple stages, enabling the model to capture both fine-grained and coarser features. This assists the model in understanding the global context and capturing local details. Finally, we employ an attention mechanism to adaptively learn the significant features within the fused representation, allowing the model to focus on the most relevant features. In summary, this module automatically fuses multi-scale features from adjacent encoder stages and, via the attention mechanism, adaptively extracts significant features from the fused representation, thereby amplifying the model’s capability to learn global and contextual multi-scale representative features.

### 3.4. Loss Function

In the training process, we employ a weighted sum of two loss functions as the final loss function. The final loss function is defined as:

(23) $$L=\lambda_{1}L_{\mathrm{ce}}+\lambda_{2}L_{\mathrm{dice}}\;$$

where $\lambda_{1}$, $\lambda_{2}$ represent two hyper-parameters that determine the relative importance of two loss functions. Experimental results indicate that the model achieves its best performance when the hyper-parameters $\lambda_{1}$ and $\lambda_{2}$ are both assigned a value of 0.5. Therefore, the values of $\lambda_{1}$ and $\lambda_{2}$ are set to 0.5 in this implementation. $L_{\mathrm{ce}}$ represents the cross-entropy loss, which serves to measure the closeness between the model’s predicted probability distribution and the true distribution. It is defined as:

(24) $$L_{\mathrm{ce}}\left(y,p\right)=-\sum_{i}y_{i}\log\left(p_{i}\right)$$

Here, $L_{\mathrm{dice}}$ represents the Dice loss function, which serves to measure the overlap between the predicted segmentation results and ground truth, and is particularly useful for handling imbalanced segmentation data. It is espressed as:

(25) $$L_{\mathrm{dice}}\left(y,p\right)=1-\frac{2\sum y_{i}p_{i}}{\sum y_{i}+\sum p_{i}}$$

where $y_{i}$ represents the true labels, $p_{i}$ represents the predicted probabilities. The term *i* denotes the *i*-th pixel.

## 4. Experiments

In this section, we primarily evaluate our method in the OCT fluid segmentation task. First, we describe the dataset used for model training and evaluation in this study. Then, we present the Implementation Details and Evaluation Metrics. Finally, we showcase the results of comparative experiments and ablation studies.

### 4.1. Datasets

We utilize the publicly available dataset: MICCAI RETOUCH challenge dataset [33]. The RETOUCH dataset is designed for segmenting three pathological areas in OCT images: intraretinal fluid (IRF), subretinal fluid (SRF), and pigment epithelial detachment (PED). This dataset comprises OCT images scanned from three devices: Zeiss Cirrus, Heidelberg Spectralis, and Topcon. The distinct differences in OCT B-scans from various devices are evident. Therefore, in this study, we conduct experiments on OCT images from each of the three devices separately. That is to say, the OCT images from the dataset are partitioned into three subsets based on the distinct acquisition devices, with each subset undergoing individual experimental analysis. Since the RETOUCH competition does not provide ground truth for the test set, we do not evaluate our model on the test set. In the comparative experiments with other methods, we employ an unbiased five-fold cross-validation method, assessing each training set from the three devices separately. For the ablation study of our model, we amalgamate the training sets from all three devices into a single dataset. On this consolidated dataset, we conducted a five-fold cross-validation to validate the efficacy of different model components. Detailed information about the dataset is illustrated in Table 1, encompassing a total of 6936 OCT training images.

### 4.2. Implementation Details and Evaluation Metrics

#### 4.2.1. Implementation Details

We apply data augmentation techniques like random flipping and random rotation to the images to enhance their diversity, preventing overfitting and boosting the model’s generalization ability. We adopt the AdamW optimizer [34] with a weight decay set to 0.01. The initial learning rate is set to 0.0001, and a “Poly” learning rate decay strategy is used throughout the training process. Our model is implemented using PyTorch and trained for 150 epochs on an NVIDIA A100 GPU. In our proposed model, the ConvNeXt is initialized using parameters pre-trained on ImageNet [35] via self-supervised learning, while the Transformer block is randomly initialized. During the training and validation phases, all OCT B-scan images from each volume in the dataset are resized to 512 × 512, with a batch size of 8. The number of the ConvNeXt blocks in CNTRB (ConvNeXt-Transformer-RASA Block) in each encoder stage is three. The number of Transformer blocks in CNTRB in each encoder stage is two. In the Transformer blocks, we use an 8 × 8 moving window, with the number of heads in each encoder stage being 3, 6, 12, and 24, respectively, increasing with the depth of the layer. As the encoder stages downsample, the number of channels in each encoder stage also changes, being 96, 192, 384, and 768, respectively.

#### 4.2.2. Evaluation Metrics

We employ the following commonly used evaluation metrics to assess the performance of our model, including Dice Similarity Coefficient (DSC), Intersection-over-Union (IoU), Relative Volume Differences (RVD), and Balanced Accuracy (BACC) [36]. Their respective definitions are as follows:

(26) $$DSC=\frac{2\times |X\cap Y|}{\left|X|+|Y\right|}$$

(27) $$IoU=\frac{\left|X\cap Y\right|}{\left|X\cup Y\right|}$$

(28) $$RVD=\frac{abs\left(|X|-|Y|\right)}{\left|Y\right|}$$

(29) $$BACC=\frac{1}{2}\left(\frac{TP}{TP+FN}+\frac{TN}{TN+FP}\right)$$

For these equations, *X* and *Y* represent the predicted segmentation result and the ground truth, respectively. |X| and |Y| represent the pixel counts of region *X* and *Y*, respectively. |X∩Y| represents the number of pixels of the intersection between |X| and |Y|. |X∪Y| represents the number of pixels of the union of |X| and |Y|. For the BACC equation, TP (True Positive) is the number of positive samples correctly classified as positive, TN (True Negative) is the number of negative samples correctly classified as negative, FP (False Positive) is the number of negative samples incorrectly classified as positive, and FN (False Negative) is the number of positive samples incorrectly classified as negative.

Both DSC and IoU are utilized to measure the degree of overlap between the predicted segmentation results and the ground truth. Their values range between 0 and 1, with values closer to 1 indicating a higher degree of overlap, thus signifying better segmentation performance. RVD represents the relative value of the volume differences between the predicted results and the ground truth. A lower value of RVD indicates a smaller discrepancy between the predicted results and the ground truth, signifying enhanced segmentation performance. BACC takes into account both the positive class and negative class recognition abilities, providing a more balanced measurement of the pixel-level classification performance of the predicted results. Through these metrics, we can evaluate the similarity between the predicted segmentation results and the ground truth, thereby assessing the model’s segmentation performance.

### 4.3. Comparisons with Other Methods

In our study, we compare the performance of our model in fluid segmentation tasks with other state-of-the-art methods, including models based on CNN and those based on Transformer. The CNN-based models include U-Net [6], U-Net++ [10], Deeplabv3+ [9], ResUnet [12], and Attention U-Net [11]. The Transformer-based models in our comparison include MsTGANet [37] Swin-UNet [27], TransUNet [26], MISSFormer [28], and H2Former [38]. These methods have been extensively applied to medical image segmentation tasks and have yielded satisfactory results. All models are trained under identical experimental settings, without any pre-processing or post-processing steps, to ensure a fair comparison. We adopt the same 5-fold cross-validation method for dataset splitting to conduct the training and validation processes, further ensuring the fairness of the comparison results. Table 2 display the quantitative comparison results of all models on the cirrus sub-dataset. The results indicate that our proposed model achieves superior performance across the majority of evaluation metrics, outperforming other competing methods. Our model improved the average Dice Similarity Coefficient (DSC) by 0.97% and 1.55% compared to TransUNet and H2Former, respectively.

As is shown in Table 3, on the Spectralis dataset, our model demonstrates an improvement of 0.81% and 1.19% in the average DSC compared to TransUNet and H2Former, respectively.

Similarly, on the Topcon dataset, as shown in Table 4, our model exhibits an increase of 0.7% and 0.85% in the average DSC when compared to TransUNet and H2Former, respectively. Our model effectively incorporates prior knowledge about the location of fluid and adaptively fuses multi-scale features from multiple encoder stages, eliminating the need for additional preprocessing steps or auxiliary information.

**Figure 4:**
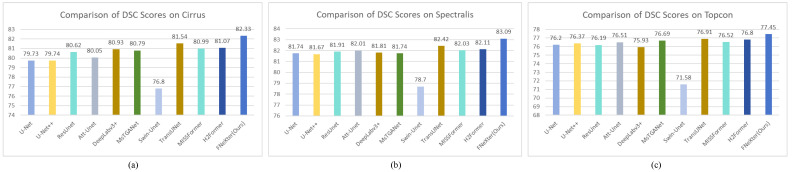
Bar chart of DSC scores for different methods on three sub-datasets. (**a**) Cirrus; (**b**) Spectralis; (**c**) Topcon.

**Figure 5:**
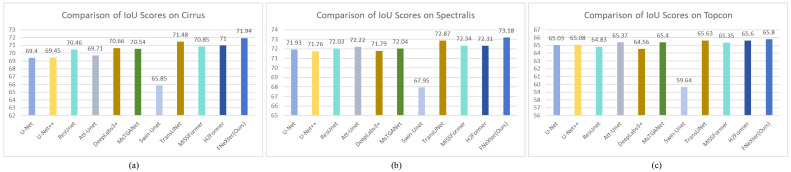
Bar chart of IoU scores for different methods on three sub-datasets. (**a**) Cirrus; (**b**) Spectralis; (**c**) Topcon.

Many studies utilized graphical methods to display comparative experiments and results, presenting the outcomes of models more effectively and intuitively [39,40]. Inspired by these studies, we have employed bar charts to exhibit the comparative results of different methods. As shown in Figure 4 and Figure 5, the visual bar charts of Dice and IoU can more clearly and intuitively demonstrate the outstanding performance of our method.

**Figure 6:**
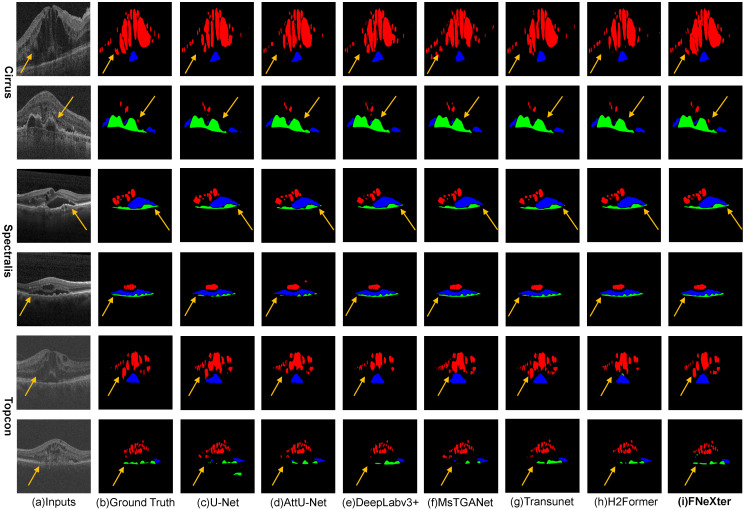
Comparison of visual segmentation results on the three Retouch sub-datasets using different methods. Red, blue, and green colors represent intraretinal fluid (IRF), subretinal fluid (SRF), and pigment epithelial detachment (PED), respectively. Our model is capable of accurately segmenting the majority of minute fluid lesions in OCT images.

Figure 6 presents the visual segmentation outcomes of several models, offering a qualitative assessment of their performances. While some models might produce segmentation inaccuracies, such as overlooking minor lesions, our model proficiently detects the majority of these subtle lesions, accurately outlining their contours and fine details.

**Figure 7:**
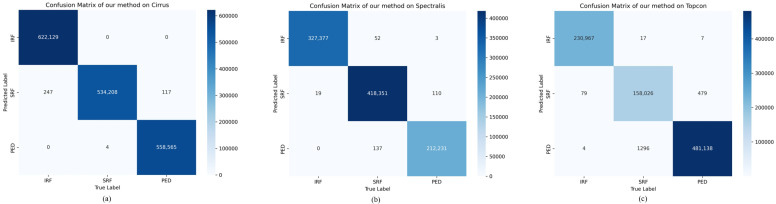
Confusion matrices of our method on different sub-datasets. (**a**) Cirrus; (**b**) Spectralis; (**c**) Topcon.

As illustrated in Figure 7, we present the confusion matrices of our method on three sub-datasets, which demonstrate the model’s segmentation performance across three categories. It proves the model’s effectiveness in segmenting lesions of different categories.

**Figure 8:**
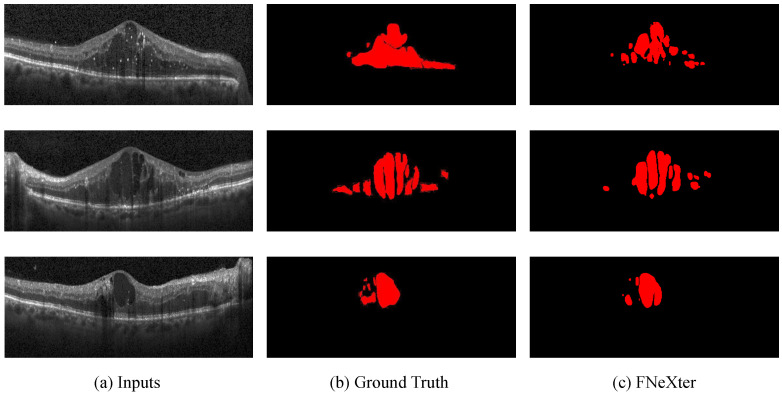
The visualized segmentation results of FNeXter on the Duke dataset.

To verify the generalization performance of our model, we conduct validation on the publicly available SD-OCT dataset of patients with diabetic macular edema (DME) from Duke University [41]. We apply our trained model, FNeXter, to the Duke dataset for inference, with the visualized segmentation results presented in Figure 8. Our model demonstrates effective segmentation of retinal fluid lesions on this dataset, achieving commendable generalization performance.

### 4.4. Ablation Studies

We amalgamate the training sets from all three devices into a single dataset. We conduct a series of ablation experiments on this consolidated dataset to validate the efficacy of our proposed model and to investigate the contribution of each component and design.

#### 4.4.1. Encoder

We conduct ablation studies to analyze the composition of the encoder, with the average segmentation results for different encoder designs reported in Table 5. Comparing various design alternatives, we observe that the model performs optimally when the encoder stage is collectively constituted by ConvNeXt, Transformer, and region-aware spatial attention (RASA), in accordance with our design. This configuration achieves the best results across all metrics, with a peak improvement of 1.2% in the average DSC. This demonstrates that the combination of ConvNeXt and Transformer blocks captures long-range dependencies and retains CNN’s inherent biases. With large convolutional kernels, our encoder efficiently encodes both local and broad contextual features across various levels and scales.

The results in Table 5 validate the role of RASA in enhancing lesion feature extraction. Furthermore, we conduct comparative experiments with other attention methods to evaluate the efficacy of the proposed RASA. Keeping other configurations constant, we compare four types of attention designs, including our proposed RASA module. The other three attention mechanisms are the Spatial Attention Module (SAM) from CBAM [42], Channel Attention (SENet) [43] and the complete CBAM (Convolutional Block Attention Module) [42]. The Table 6 reports the segmentation results when employing each of these four attention modules. Among them, the model incorporating our RASA design yields the best performance across all metrics. These findings indicate that standard attention mechanisms fail to introduce lesion location prior knowledge into the model, leading to insufficient focus on spatial features. In contrast, RASA effectively integrates prior knowledge about lesion locations into the model, enhancing the model’s sensitivity to lesions. This integration also bolsters the extraction of multi-scale spatial features, subsequently improving segmentation results.

#### 4.4.2. Decoder

We conduct ablation studies to assess the implications of the decoder’s design. Keeping other configurations constant, we alter only the design of the decoder for comparison. We use the decoder from U-Net [6] as the baseline and compare scenarios where ConvNeXt and Transformer are used individually as well as in combination for constructing the decoder. The results, as indicated in Table 7, show that the model performs optimally when the decoder is solely composed of Transformer blocks. This can be attributed to the Transformer’s inherent ability to capture long-range dependencies and non-local self-similarity. The window-based multi-head self-attention mechanism of the Transformer allows it to relate and weigh features across different positions, which is crucial during the decoding process to understand and reconstruct complex structures and patterns in the images.

#### 4.4.3. Self-Adaptive Multi-Scale Feature Fusion Attention (SMFFA)

We conducted an ablation study to compare our proposed SMFFA module with the conventional skip connections, the results of which are presented in Table 8. Traditional skip connections, as employed in methods like U-Net, concatenate the features from the encoder stage directly with the corresponding features from the decoder stage. The findings indicate that using SMFFA yields better performance than using conventional skip connections, thereby validating the effectiveness of the SMFFA module. This is attributed to the effective fusion of multi-scale, multi-stage, and multi-level features by concatenating the multi-scale features from the current encoder stage with those from adjacent encoder stages. This concatenated representation feature then undergoes an attention module to further self-adaptively augment the fused features. As a result, the enhanced skip connections provide the model with more effective global features and multi-scale contextual information.

## 5. Conclusions

In this paper, we propose a novel FNeXter network aimed at enhancing the accuracy of fluid segmentation. Within the FNeXter architecture, we incorporate a feature extraction module based on a hybrid of ConvNeXt and Transformer, complemented by the RASA and SMFFA modules. The Transformer is adept at modeling long-range dependencies and non-local similarities. In contrast, ConvNeXt retains the inductive bias intrinsic to CNNs and excels in extracting detailed information from localized regions; its large convolutional kernels further aid in capturing broader contextual insights. The RASA module incorporates prior knowledge about fluid locations, steering the model’s focus towards the central areas where lesions predominantly occur, thereby enhancing the model’s sensitivity to lesion-specific spatial features. The SMFFA module improves the model’s ability to learn global features and multi-scale contextual information by fusing and extracting multi-level, multi-scale features from adjacent encoder stages. With these integrative designs, we significantly bolster the accuracy and robustness of fluid segmentation in retinal OCT images. Our model achieves state-of-the-art segmentation results across three RETOUCH sub-datasets originating from distinct devices.

Moving forward, we intend to employ self-supervised techniques for pre-training on OCT images, aiming to bridge the domain gap between the pre-trained model and the target segmentation task.

## Figures and Tables

**Table 1:** Overview of Retouch dataset.

Type	Cirrus	Spectralis	Topcon
Volume size	512 × 1024 × 128	512 × 496 × 49	512 × 650/885 × 128/64
Training (V/S)	24/3072	24/1176	22/2688
Test (V/S) *	14/860	14/430	14/1004

V = Volumes, S = Slices, * The ground truth of test set is not available.

**Table 2:** Quantitative comparisons with state-of-the-art methods on the retouch sub-dataset Cirru s (5-fold cross-validation).

Method	DSC	IoU	AVD	BACC
U-Net [6]	79.73 ± 0.59	69.40 ± 0.71	19.46 ± 1.75	93.84 ± 0.29
U-Net++ [10]	79.74 ± 0.69	69.45 ± 0.88	18.72 ± 1.40	93.91 ± 0.34
ResUnet [12]	80.62 ± 0.57	70.46 ± 0.71	17.79 ± 1.32	94.17 ± 0.38
Att-UNet [11]	80.05 ± 0.56	69.71 ± 0.74	19.27 ± 1.92	93.91 ± 0.17
DeepLabv3+ [9]	80.93 ± 0.86	70.66 ± 1.05	17.84 ± 0.97	94.33 ± 0.40
MsTGANet [37]	80.79 ± 0.60	70.54 ± 0.69	18.63 ± 1.42	94.36 ± 0.35
Swin-UNet [27]	76.80 ± 0.56	65.85 ± 0.68	20.21 ± 1.14	93.35 ± 0.38
TransUNet [26]	81.54 ± 0.59	71.48 ± 0.69	18.48 ± 1.89	94.52 ± 0.42
MISSFormer [28]	80.99 ± 0.55	70.85 ± 0.71	18.69 ± 1.45	94.28 ± 0.39
H2Former [38]	81.07 ± 0.60	71.00 ± 0.74	18.55 ± 1.71	94.61 ± 0.20
FNeXter (Ours)	**82.33 ± 0.46**	**71.94 ± 0.61**	**16.32 ± 1.33**	**94.83 ± 0.25**

Bold indicates the best.

**Table 3:** Quantitative comparisons with state-of-the-art methods on the retouch sub-dataset Spectrali s (5-fold cross-validation).

Method	DSC	IoU	AVD	BACC
U-Net [6]	81.74 ± 0.67	71.93 ± 0.87	17.52 ± 1.28	94.08 ± 0.28
U-Net++ [10]	81.67 ± 1.29	71.76 ± 1.44	18.52 ± 2.51	94.14 ± 0.39
ResUnet [12]	81.91 ± 0.52	72.03 ± 0.71	17.58 ± 1.76	94.09 ± 0.18
Att-UNet [11]	82.01 ± 0.76	72.22 ± 0.95	16.76 ± 1.14	94.11 ± 0.33
DeepLabv3+ [9]	81.81 ± 1.12	71.79 ± 1.19	17.76 ± 3.38	94.03 ± 0.22
MsTGANet [37]	81.74 ± 0.84	72.04 ± 1.01	16.99 ± 1.89	93.93 ± 0.20
Swin-UNet [27]	78.70 ± 0.93	67.95 ± 1.20	21.62 ± 3.48	93.03 ± 0.56
TransUNet [26]	82.42 ± 0.71	72.87 ± 0.83	17.98 ± 2.72	94.22 ± 0.10
MISSFormer [28]	82.03 ± 0.95	72.34 ± 1.11	19.47 ± 3.66	94.18 ± 0.21
H2Former [38]	82.11 ± 1.00	72.31 ± 1.20	20.40 ± 4.52	94.67 ± 0.22
FNeXter (Ours)	**83.09 ± 0.93**	**73.18 ± 1.21**	**16.48 ± 1.67**	**94.69 ± 0.37**

Bold indicates the best.

**Table 4:** Quantitative comparisons with state-of-the-art methods on the retouch sub-dataset Topco n (5-fold cross-validation).

Method	DSC	IoU	AVD	BACC
U-Net [6]	76.20 ± 1.21	65.03 ± 1.21	26.12 ± 3.03	92.64 ± 0.37
U-Net++ [10]	76.37 ± 0.80	65.08 ± 0.84	30.56 ± 6.69	92.80 ± 0.47
ResUnet [12]	76.19 ± 0.65	64.83 ± 0.80	27.38 ± 4.56	92.95 ± 0.72
Att-UNet [11]	76.51 ± 0.98	65.37 ± 1.05	25.17 ± 2.69	92.59 ± 0.33
DeepLabv3+ [9]	75.93 ± 0.96	64.56 ± 0.92	23.75 ± 2.26	92.26 ± 0.28
MsTGANet [37]	76.69 ± 0.71	65.40 ± 0.79	25.49 ± 2.13	92.64 ± 0.25
Swin-UNet [27]	71.58 ± 0.72	59.64 ± 0.67	28.49 ± 2.23	91.28 ± 0.39
TransUNet [26]	76.91 ± 0.64	65.63 ± 0.58	26.03 ± 2.86	93.09 ± 0.36
MISSFormer [28]	76.52 ± 0.73	65.35 ± 0.61	25.87 ± 2.19	92.89 ± 0.32
H2Former [38]	76.80 ± 0.65	65.60 ± 0.69	25.27 ± 1.77	93.00 ± 0.39
FNeXter (Ours)	**77.45 ± 0.60**	**65.80 ± 0.56**	**23.54 ± 1.70**	**93.32 ± 0.56**

Bold indicates the best.

**Table 5:** Ablation studies of encoder stage components.

ConvNeXt	Transformer	RASA	DSC	IoU	AVD	BACC
✓			79.57	69.20	19.97	93.98
	✓		79.25	68.77	20.39	94.13
✓	✓		79.80	69.44	19.52	93.98
✓	✓	✓	**80.20**	**69.85**	**18.66**	**94.20**

Bold indicates the best.

**Table 6:** Performance comparisons of different attention methods.

Method	DSC	IoU	AVD	BACC
SAM [42]	79.85	69.53	19.60	94.12
SENet [43]	79.86	69.55	19.34	93.99
CBAM [42]	79.98	69.65	18.82	94.13
RASA	**80.20**	**69.85**	**18.66**	**94.20**

Bold indicates the best.

**Table 7:** Ablation studies of decoder components.

Decoder	DSC	IoU	AVD	BACC
U-Net Decoder [6]	79.74	69.35	19.21	93.85
Transformer	**80.20**	**69.85**	**18.66**	**94.20**
ConvNeXt	79.78	69.39	19.23	93.91
ConvNeXt + Transformer	79.86	69.46	18.73	93.86

Bold indicates the best.

**Table 8:** Ablation studies of SMFFA module.

Method	DSC	IoU	AVD	BACC
w/o SMFFA	79.53	69.25	19.32	93.99
w/ SMFFA	**80.20**	**69.85**	**18.66**	**94.20**

Bold indicates the best.

## Data Availability

The datasets in the paper are publicly available.

## References

[1] Bhagat N., Grigorian R.A., Tutela A., Zarbin M.A. (2009). Diabetic macular edema: Pathogenesis and treatment. Surv. Ophthalmol..

[2] Huang D., Swanson E.A., Lin C.P., Schuman J.S., Stinson W.G., Chang W., Hee M.R., Flotte T., Gregory K., Puliafito C.A. (1991). Optical coherence tomography. Science.

[3] Zhang M., Wang J., Pechauer A.D., Hwang T.S., Gao S.S., Liu L., Liu L., Bailey S.T., Wilson D.J., Huang D. (2015). Advanced image processing for optical coherence tomographic angiography of macular diseases. Biomed. Opt. Express.

[4] Wu M., Chen Q., He X., Li P., Fan W., Yuan S., Park H. (2017). Automatic subretinal fluid segmentation of retinal SD-OCT images with neurosensory retinal detachment guided by enface fundus imaging. IEEE Trans. Biomed. Eng..

[5] Montuoro A., Waldstein S.M., Gerendas B.S., Schmidt-Erfurth U., Bogunović H. (2017). Joint retinal layer and fluid segmentation in OCT scans of eyes with severe macular edema using unsupervised representation and auto-context. Biomed. Opt. Express.

[6] Ronneberger O., Fischer P., Brox T. (2015). U-net: Convolutional networks for biomedical image segmentation. Medical Image Computing and Computer-Assisted Intervention, Proceedings of the Medical Image Computing and Computer-Assisted Intervention–MICCAI 2015: 18th International Conference, Munich, Germany, 5–9 October 2015.

[7] Long J., Shelhamer E., Darrell T. Fully convolutional networks for semantic segmentation. Proceedings of the IEEE Conference on Computer Vision and Pattern Recognition.

[8] Badrinarayanan V., Kendall A., Cipolla R. (2017). Segnet: A deep convolutional encoder-decoder architecture for image segmentation. IEEE Trans. Pattern Anal. Mach. Intell..

[9] Chen L.C., Zhu Y., Papandreou G., Schroff F., Adam H. Encoder-decoder with atrous separable convolution for semantic image segmentation. Proceedings of the European Conference on Computer Vision (ECCV).

[10] Zhou Z., Siddiquee M.M.R., Tajbakhsh N., Liang J. (2019). Unet++: Redesigning skip connections to exploit multiscale features in image segmentation. IEEE Trans. Med. Imaging.

[11] Oktay O., Schlemper J., Folgoc L.L., Lee M., Heinrich M., Misawa K., Mori K., McDonagh S., Hammerla N.Y., Kainz B. (2018). Attention u-net: Learning where to look for the pancreas. arXiv.

[12] Zhang Z., Liu Q., Wang Y. (2018). Road extraction by deep residual u-net. IEEE Geosci. Remote Sens. Lett..

[13] Isensee F., Jaeger P.F., Kohl S.A., Petersen J., Maier-Hein K.H. (2021). nnU-Net: A self-configuring method for deep learning-based biomedical image segmentation. Nat. Methods.

[14] Lu D., Heisler M., Lee S., Ding G.W., Navajas E., Sarunic M.V., Beg M.F. (2019). Deep-learning based multiclass retinal fluid segmentation and detection in optical coherence tomography images using a fully convolutional neural network. Med. Image Anal..

[15] Zhu W., Zhang L., Shi F., Xiang D., Wang L., Guo J., Yang X., Chen H., Chen X. (2017). Automated framework for intraretinal cystoid macular edema segmentation in three-dimensional optical coherence tomography images with macular hole. J. Biomed. Opt..

[16] Gopinath K., Sivaswamy J. (2018). Segmentation of retinal cysts from optical coherence tomography volumes via selective enhancement. IEEE J. Biomed. Health Inform..

[17] Hassan T., Akram M.U., Masood M.F., Yasin U. (2019). Deep structure tensor graph search framework for automated extraction and characterization of retinal layers and fluid pathology in retinal SD-OCT scans. Comput. Biol. Med..

[18] Hu J., Chen Y., Yi Z. (2019). Automated segmentation of macular edema in OCT using deep neural networks. Med. Image Anal..

[19] Feng S., Zhao H., Shi F., Cheng X., Wang M., Ma Y., Xiang D., Zhu W., Chen X. (2020). CPFNet: Context pyramid fusion network for medical image segmentation. IEEE Trans. Med. Imaging.

[20] Liu X., Wang S., Zhang Y., Liu D., Hu W. (2021). Automatic fluid segmentation in retinal optical coherence tomography images using attention based deep learning. Neurocomputing.

[21] Xing G., Chen L., Wang H., Zhang J., Sun D., Xu F., Lei J., Xu X. (2022). Multi-scale pathological fluid segmentation in OCT with a novel curvature loss in convolutional neural network. IEEE Trans. Med. Imaging.

[22] Deng Z., Cai Y., Chen L., Gong Z., Bao Q., Yao X., Fang D., Yang W., Zhang S., Ma L. (2022). Rformer: Transformer-based generative adversarial network for real fundus image restoration on a new clinical benchmark. IEEE J. Biomed. Health Inform..

[23] Xie E., Wang W., Yu Z., Anandkumar A., Alvarez J.M., Luo P. (2021). SegFormer: Simple and efficient design for semantic segmentation with transformers. Adv. Neural Inf. Process. Syst..

[24] Dosovitskiy A., Beyer L., Kolesnikov A., Weissenborn D., Zhai X., Unterthiner T., Dehghani M., Minderer M., Heigold G., Gelly S. (2020). An image is worth 16x16 words: Transformers for image recognition at scale. arXiv.

[25] Liu Z., Lin Y., Cao Y., Hu H., Wei Y., Zhang Z., Lin S., Guo B. Swin transformer: Hierarchical vision transformer using shifted windows. Proceedings of the IEEE/CVF International Conference on Computer Vision.

[26] Chen J., Lu Y., Yu Q., Luo X., Adeli E., Wang Y., Lu L., Yuille A.L., Zhou Y. (2021). Transunet: Transformers make strong encoders for medical image segmentation. arXiv.

[27] Cao H., Wang Y., Chen J., Jiang D., Zhang X., Tian Q., Wang M. (2022). Swin-unet: Unet-like pure transformer for medical image segmentation. Computer Vision, Proceedings of the European Conference on Computer Vision, Tel Aviv, Israel, 23–27 October 2022.

[28] Huang X., Deng Z., Li D., Yuan X., Fu Y. (2023). Missformer: An effective transformer for 2d medical image segmentation. IEEE Trans. Med. Imaging.

[29] Wang H., Cao P., Wang J., Zaiane O.R. Uctransnet: Rethinking the skip connections in u-net from a channel-wise perspective with transformer. Proceedings of the AAAI Conference on Artificial Intelligence.

[30] Liu Z., Mao H., Wu C.Y., Feichtenhofer C., Darrell T., Xie S. A convnet for the 2020s. Proceedings of the IEEE/CVF conference on Computer Vision and Pattern Recognition.

[31] Woo S., Debnath S., Hu R., Chen X., Liu Z., Kweon I.S., Xie S. Convnext v2: Co-designing and scaling convnets with masked autoencoders. Proceedings of the IEEE/CVF Conference on Computer Vision and Pattern Recognition.

[32] Tao H., Duan Q. (2024). Hierarchical attention network with progressive feature fusion for facial expression recognition. Neural Netw..

[33] Bogunović H., Venhuizen F., Klimscha S., Apostolopoulos S., Bab-Hadiashar A., Bagci U., Beg M.F., Bekalo L., Chen Q., Ciller C. (2019). RETOUCH: The retinal OCT fluid detection and segmentation benchmark and challenge. IEEE Trans. Med. Imaging.

[34] Loshchilov I., Hutter F. Decoupled Weight Decay Regularization. Proceedings of the International Conference on Learning Representations.

[35] Deng J., Dong W., Socher R., Li L.J., Li K., Fei-Fei L. Imagenet: A large-scale hierarchical image database. Proceedings of the 2009 IEEE Conference on Computer Vision and Pattern Recognition.

[36] Brodersen K.H., Ong C.S., Stephan K.E., Buhmann J.M. The balanced accuracy and its posterior distribution. Proceedings of the 2010 20th International Conference on Pattern Recognition.

[37] Wang M., Zhu W., Shi F., Su J., Chen H., Yu K., Zhou Y., Peng Y., Chen Z., Chen X. (2021). MsTGANet: Automatic drusen segmentation from retinal OCT images. IEEE Trans. Med. Imaging.

[38] He A., Wang K., Li T., Du C., Xia S., Fu H. (2023). H2Former: An Efficient Hierarchical Hybrid Transformer for Medical Image Segmentation. IEEE Trans. Med. Imaging.

[39] Khan Y.D., Khan N.S., Naseer S., Butt A.H. (2021). iSUMOK-PseAAC: Prediction of lysine sumoylation sites using statistical moments and Chou’s PseAAC. PeerJ.

[40] Liu T., Huang J., Luo D., Ren L., Ning L., Huang J., Lin H., Zhang Y. (2024). Cm-siRPred: Predicting chemically modified siRNA efficiency based on multi-view learning strategy. Int. J. Biol. Macromol..

[41] Chiu S.J., Allingham M.J., Mettu P.S., Cousins S.W., Izatt J.A., Farsiu S. (2015). Kernel regression based segmentation of optical coherence tomography images with diabetic macular edema. Biomed. Opt. Express.

[42] Woo S., Park J., Lee J.Y., Kweon I.S. (2018). Cbam: Convolutional block attention module. Computer Vision, Proceedings of the European Conference on Computer Vision (ECCV), Munich, Germany, 8–14 September 2018.

[43] Hu J., Shen L., Sun G. Squeeze-and-excitation networks. Proceedings of the IEEE Conference on Computer Vision and Pattern Recognition.

